# Potential Effects of Delphinidin-3-*O*-Sambubioside and Cyanidin-3-*O*-Sambubioside of *Hibiscus sabdariffa* L. on Ruminant Meat and Milk Quality

**DOI:** 10.3390/ani11102827

**Published:** 2021-09-28

**Authors:** Rosalba Lazalde-Cruz, Luis Alberto Miranda-Romero, Deli Nazmín Tirado-González, María Isabel Carrillo-Díaz, Sergio Ernesto Medina-Cuéllar, Germán David Mendoza-Martínez, Alejandro Lara-Bueno, Gustavo Tirado-Estrada, Abdelfattah Z. M. Salem

**Affiliations:** 1 Posgrado en Producción Animal, Departamento de Zootecnia, Universidad Autónoma Chapingo, Carretera México-Texcoco km 38.5, Texcoco 56230, Estado de México, Mexico; rlazaldc.909@gmail.com (R.L.-C.); microbiologia.pecuaria08@gmail.com (L.A.M.-R.); alarab_11@hotmail.com (A.L.-B.); 2 Centro Nacional de Investigación Disciplinaria Agricultura Familiar (CENID AF), Instituto Nacional de Investigaciones Forestales, Agrícolas y Pecuarias (INIFAP), Carretera Lagos de Moreno-Jalisco km 8.5, Ojuelos de Jalisco 47540, Jalisco, Mexico; 3 Facultad de Medicina Veterinaria y Zootecnia, Universidad de Colima, Autopista Colima-Manzanillo km 40, Tecomán 28100, Colima, Mexico; mcarrillo13@ucol.mx; 4 Departamento de Arte y Empresa, División de Ingenierías Campus Irapuato-Salamanca, Universidad de Guanajuato, Carretera Salamanca-Valle de Santiago km 3.5 + 1.8, Salamanca 36885, Guanajuato, Mexico; se.medina@ugto.mx; 5 Facultad de Medicina Veterinaria y Zootecnia, Universidad Autónoma de México-Xochimilco, Calzada del Hueso 1100, Coapa, Villa Quietud, Coyoacán 04960, Ciudad de Mexico, Mexico; gmendoza@correo.xoc.uam.mx; 6 Instituto Tecnológico El Llano Aguascalientes (ITEL), Tecnológico Nacional de México (TecNM), Carretera Aguascalientes-S.L.P. km 18.5, El Llano 20330, Aguascalientes, Mexico; 7 Facultad de Veterinaria y Zootecnia, Universidad Autónoma del Estado de México, Toluca 50295, Estado de Mexico, Mexico

**Keywords:** anthocyanins, cyanidin-3-*O*-sambubioside, delphinidin-3-*O*-sambubioside, *Hibiscus sabdariffa* L., fat milk and meat quality, milk and meat production, ruminant nutrition

## Abstract

**Simple Summary:**

*Hibiscus sabdariffa* (HS) calyxes are widely used as nutraceutical supplements in humans; however, stalks, leaves, and seeds are considered as agriculture by-products. Including HS by-products in animal feeding could reduce economic costs and environmental problems, and due to their bioactive compounds, could even improve the quality of meat and milk. However, although HS antioxidants have not been tested enough in ruminants, comparison with other by-products rich in polyphenols allows for hypothesizing on the potential effects of including HS by-products and calyxes in nutrition, animal performance, and meat and milk quality. Antioxidants of HS might affect ruminal fiber degradability, fermentation patterns, fatty acids biohydrogenation (BH), and reduce the methane emissions. After antioxidants cross into the bloodstream and deposit into ruminants’ milk and meat, they increase the quality of fatty acids, the antioxidant activity, and the shelf-life stability of dairy products and meat, which leads to positive effects in consumers’ health. In other animals, the specific anthocyanins of HS have improved blood pressure, which leads to positive clinical and chemicals effects, and those could affect some productive variables in ruminants. The HS by-products rich in polyphenols and anthocyanins can improve fatty acid quality and reduce the oxidative effects on the color, odor, and flavor of milk products and meat.

**Abstract:**

The objective was to review the potential effects of adding anthocyanin delphinidin-3-*O*-sambubioside (DOS) and cyanidin-3-*O*-sambubioside (COS) of HS in animal diets. One hundred and four scientific articles published before 2021 in clinics, pharmacology, nutrition, and animal production were included. The grains/concentrate, metabolic exigency, and caloric stress contribute to increasing the reactive oxygen species (ROS). COS and DOS have antioxidant, antibacterial, antiviral, and anthelmintic activities. In the rumen, anthocyanin might obtain interactions and/or synergisms with substrates, microorganisms, and enzymes which could affect the fiber degradability and decrease potential methane (CH_4_) emissions; since anthocyanin interferes with ruminal fatty acids biohydrogenation (BH), they can increase the n-3 and n-6 polyunsaturated fatty acids (PUFA), linoleic acid (LA), and conjugated linoleic acid (CLA) in milk and meat, as well as improving their quality. Anthocyanins reduce plasma oxidation and can be deposited in milk and meat, increasing antioxidant activities. Therefore, the reduction of the oxidation of fats and proteins improves shelf-life. Although studies in ruminants are required, COS and DOS act as inhibitors of the angiotensin-converting enzyme (ACEi) and rennin expression, regulating the homeostatic control and possibly the milk yield and body weight. By-products of HS contain polyphenols as calyces with positive effects on the average daily gain and fat meat quality.

## 1. Introduction

*Hibiscus sabdariffa* L. (HS) is a type of shrub of *Malvaceae* family from India [1,2], adapted to spring–summer and subtropical or tropical environments (Aw/As—Köppen climate classification) [2,3,4]. In Mexico, HS production has increased 10.54% from 2003 to 2018 [5,6] of shrubs of the *Malvaceae* family from India [1,2], adapted to spring–summer and subtropical or tropical environments (Aw/As—Köppen climate classification) [2,3,4].

According to FAO [7], the HS calyxes are one of the most demanded products by industry for human feeding [8,9]. Due to fatty acids, HS contents and proportions [10,11], antioxidants [12,13,14], antimicrobial (Gramm negative bacteria) [15], antiviral [16], and anthelmintic properties [17], might lead to improvements in human health [12,18,19].

Calyxes of HS contain 15.76–0.04% of a linoleic fatty acid (n-3) [10,11] and flavonoids classified as anthocyanins [20]. Factors such as the HS type variety, crop management, processing, storage, extraction of extracts, and cell contents affect the antioxidant contents [21,22,23]. However, the highest proportions of HS flavonoids are the anthocyanins cyanidid-3-*O*-sambubioside (COS) (25.9 to 46.2%) and delphinidin-3-*O*-sambubioside (DOS) (48.4 to 59.2%) [13,15,23,24], whose clinical effects on humans are different from other kinds of flavonoids supplements such as green teas (*Camelia sinensis*), which mainly contain epigalocathechin-3-gallate (EGCG), epigallocatechin (EGC), epicahechin-3-gallate (ECG), and epicatechin (EC) [25].

The leaves, stalks, and seeds of HS, as well as other agro-industrial residues, can be used to feed livestock and reduce environmental impact and production costs. Moreover, their phytochemicals such as polyphenols and vitamins could improve the meat and milk quality as well as their shelf-life stability [17,26,27].

Overall, polyphenols and other kinds of antioxidants such as selenium and a-tocopherol reduce the free radicals and chelate pro-oxidant metals [9,14,28,29,30] and can affect the ruminal digestibility and fermentation kinetics [31,32,33], as well as in animal productive behavior [33], reducing the effects of the oxidative stress in ruminants [34,35,36] caused by the high grain and concentrate proportions on diets [25,37,38], the metabolic exigency, and the heat stress [38], and therefore also improve the oxide-reductive potential of products derived for human feed [39].

The objective of the present study was to conduct a critical review about the potential effects of HS anthocyanins, COS and DOS, on ruminant diets, meat and milk quality, and their shelf-life stability.

## 2. *Hibiscus sabdariffa* L. By-Products

**Figure 1:**
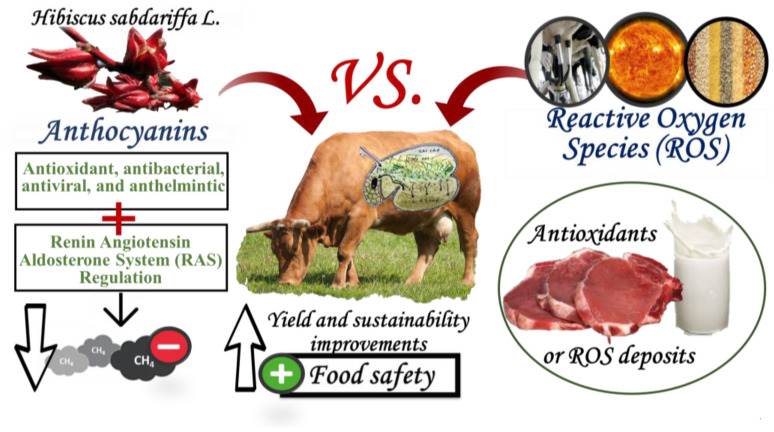
Overall economic, environmental, and productive effects of including *Hibsicus sabdariffa* L. calyxes and by-products in ruminant diets.

Figure 1 summarizes how the agricultural wastes and by-products of HS such as seeds, stalks, and leaves can reduce the economic and environmental livestock costs [40,41]. Additionally, optimal inclusion of by-products and wastes of HS in balanced ruminant diets should not have negative effects on animal productive behavior [42,43,44,45] but rather improve the meat and milk quality.

The phenolic and antioxidant activities of HS seeds have been previously assayed, and results were similar or better than those in calyxes [46]. However, the comparison of the potential effects of seeds with calyxes should be assayed in ruminal liquid, including a test to interpret the ruminal microorganisms, fibrolytic enzymes, with the feedstuff’s cell walls.

Table 1 shows the chemical composition of HS seeds reported by different authors, and on average, HS seeds contain: crude protein (CP), 27.9 ± 10 g/100 g of dry matter (DM); fat, 18.8 ± 8.6 g/100 g DM; crude fiber (CF), 16.8 ± 11.1 g/100 DM; and ashes, 5.86 ± 3.2 g/100 DM [43,47].

The CP content of HS seeds is comparable to soybean and canola seeds [59], as their fatty acids are primarily oleic and linoleic acids (LA) (n-9 and n-6 polyunsaturated fatty acids (PUFA)) (37.68 ± 1.10% and 34.14 ± 1.25%); however, calyxes contains similar contents of LA and more α-linoleic acids than seeds (LA: 34.14% vs. 32.65%, and α-linoleic: 1.77% vs. 15.76%, seeds vs. calyxes) (Table 2) [10,58,60], and its DM and CP in situ degradability had been similar to sunflower and peanut seeds [61].

## 3. Oxidative Stress in Ruminants

Inflammatory and environmental processes increase the endogenous reactive oxygen species (ROS). Unbalance between pro-oxidants and antioxidants might promote oxidative stress and molecular damage [38].

In dairy cows and beef cattle, the environmental pollution and the high metabolic exigency during pregnancy, milk production, heat stress, respiratory diseases, inflammatory process, and parasites promote ROS releasing (O_2_, OH, RO_2_, RO, HO_2_, H_2_O_2_, HOCl, O_3_, etc.); meanwhile, the adipose mobilization increases the pro-inflammatory cytokines [38]. The potential negative effects on animal wealth would also worsen in the future because of the population increment and therefore the milk and meat demand [28].

The ROS contribute to inflammatory processes through necroptosis activation (NF-κβ) via phosphorylation interleukin (I-κβ) and because of the production of pro-inflammatory cytokines such as tumoral factors (TNF-α). In addition, protein carbonylation is mediated by the ROS and metals (Fe^2+^, Cu^+^, etc.), producing oxidative by-products and advanced oxidative protein products (AOPP): (1) carbohydrates and lipids have reactive compounds to carbonyl from glycoxidation and lipoperoxidation that might bond to protein residues; (2) oxidized proteins are degraded by proteases, but chemically modified proteins (by di-tyrosine and disulfide cross linkages) might not be substrates to proteolysis, contributing to deposits in tissues and organ damages [62,63,64,65,66,67].

High grain and concentrate proportions in ruminant diets increases lipoperoxidation, decreasing the α-tocopherol and the ferric reductive availability in blood plasma [22] and increasing the amount of AOPP, which is negatively related with milk yield because of the oxide-reductive unbalance. Including high-grain diets and therefore the reduction of forage proportion rises the abnormal amount and types of metabolites in rumen [38].

In viral, bacterial, and fungal infections, phagocytes and neutrophils are sources of ROS that interfere in a chain of chemical reactions which increase the hypochlorous oxidant potential and which might be useful to combat the photogenes, but while also damaging tissues. Besides this, parasites induce inflammation followed by an increase in eosinophils, which also contribute to tissue damage. Lactation and heat stress are potential sources of AOPP and thereby of TNF-α expression and potential mammal glandule diseases, increasing the milk and meat contents of ROS [68,69].

Oxidized milk and meat contribute to a higher ROS content in blood plasma which would be a threat to human health [39].

## 4. Potential Clinic Effects of Antioxidants

Polyphenols are a wide variety of secondary plant metabolites with at least one -OH that can be structurally simple (egallic and gallic acids) or complex (dimers, oligomeric, and polymeric with high molecular weight). Antioxidants can be classified as flavonoids or non-flavonoids. Thus, flavonoids can be flavones, flavanones, isoflavones, flavonols, flavan-3-ols; on the other hand, anthocyanins (from flavan-3-ols derived the condensed tannins (non-hydrolysable)), phenolic acids, hydrolysable tannins, and stilbenes are clustered as non-flavonoids [17].

Because of the structural differences among complexes, total phenolic compounds cannot directly be related with total antioxidant availability [21,22]. EGCG, primarily found in green tea, was found to have a galloyl group in the third position and an o-trihydroxy in the β-ring which protects cells from ROS damage [64,70]; by the regulation the overexpression of genes, EGCG has anti-inflammatory and antioxidant effects in reduction of apoptosis, cell fibrosis, and tumoral growing via regulation and reduction of kinases, signal transduction, and transcription activation [66,71]. EGCG can [72]:

(1) Promote the cytotoxicity to increase the antitumoral activities by producing H_2_O_2_ with its pyrogallol moiety or the reduction of Fe(III) to Fe(II), generating -OH ROS (although cysteine N-acetyl protect cells from cytotoxicity of H_2_O_2_, it does not avoid cell death process).
(2) Promote apoptosis through mitochondrial damage, membrane depolarization, and cytochrome c release, and protects against mitochondrial damage-related cell death without changes in superoxide dismutase (SOD), glutathione peroxidase, Nrf2, Bcl2, and oxidative stress. Modulates gene expression by inhibiting various transcription factors (including Sp1, NF-κB, AP-1, STAT1, STAT3, and FOXO1) and the expression of NF-κB and AP-1. EGCG inhibits STAT1 to mediate protective effects on myocardial injury.
(3) Increase second messengers, such as Ca^2+^, cAMP, and cGMP. EGCG elevates cytosolic Ca^2+^ without electrical stimulation by inhibition of sarcoplasmic/endoplasmic reticulum Ca^2+^-ATPase activity (SERCA), which affects the activities of Ca^2+^-requiring enzymes, such as calmodulin (CAM)-dependent protein kinase II and CAMKKβ (CAMKKβ is an upstream regulator of AMP-dependent kinase (AMPK), which plays crucial roles in energy metabolism and cardiovascular functions). If it stimulates vasorelaxation by increasing cAMP and cGMP in the aorta, then it may stimulate the production of cyclic nucleotides with beneficial biological effects in cardiovascular physiology.
(4) Inhibit the transcription of FOXO1 to lead to the suppression of basal levels of endothelin-1 and differentiation of adipocytes. In mitochondria, EGCG enhances fat utilization, reducing the expression of leptin and stearyl-CoA desaturase while increasing fat oxidation. Moreover, EGCG regulates activities of cell surface growth factor receptors, especially receptor tyrosine kinases (RTK), including epidermal growth factor receptor (EGFR), vascular endothelial growth factor receptor (VEGFR), insulin-like growth factor receptor (IGFR), and the insulin receptor (InsR).
(5) Inhibit DNA methyltransferase, which reverses methylation-induced gene silencing.
(6) Inhibit autophagy, leading to apoptosis in macrophage cell lines.

Although the extracts of HS also change the oxidative potential of blood plasma, increasing the glutathione intracellular, its primarily action is on the Renin–Angiotensin–Aldosterone System (RAS) interfering with electrolytic regulation, blood pressure, and cardiac function [73], as well as the increasing of adrenalin, catecholamines, and noradrenalin (by specifically angiotensin (AngII)) [74].

Guerrero et al. [75] tested the activity of the Angiotensin Converting Enzyme inhibitor (ACEi) of 17 different types of flavonoids, and the ACEi increased when: (1) the catechol group was in the β-ring (3’, 4’-dihidroxy); (2) there is a double bond between C2 and C3 of carbon rings; and (3) there is a ketone in the C4 of the carbon ring. The absence of C4 in the carbonyl group of EGCG reduces the ACEi ability; the DOS and COS chemical structures have primarily ACEi potential.

Studies including in vivo cells [73] have shown that DOS and COS inhibit 43 to 50% of the ACE (COS and COS vs. control, and 30% less than captopril); furthermore, anthocyanins interfered in the RAS reductive process (RT-qPCR mARN of ACE and renin were analyzed), reducing 37 to 52% of the rARN expression for renin. To test the clinical effect of anthocyanins of HS, Nurfaradilla et al. [76] blocked the left renal artery of mice (2KlC hypertension) and treated them with HS extracts (30 mg/200 g BW), captopril, and captopril+HS mixtures; HS extracts reduced the systolic blood pressure 17% (average 150 vs. 88, and 80, control vs. HS, and captopril). Although captopril and HS reduced the renin and AngII in plasma, HS reduced the ACE activity (1.5 µmol/mL/min control vs. 0.40 µmol/mL/min HS, vs. 0.30 µmol/mL/min captopril).

Other potential pharmacological properties of HS antioxidants are anti-hypercholesterolemia, antipyretic, antibacterial, antiviral, and anthelminthic [13,77].

## 5. Effect of Anthocyanins on Diet Nutritive Value and Productive Behavior in Ruminants

### 5.1. Effects on Ruminal Digestibility, Volatile Fatty Acids, and Methane Emission

Antioxidants might maintain their activities in a ruminal environment and reach to the bowel without major modifications. Anthocyanins can improve the ruminal antioxidant potential [31,32].

Although some in vitro studies show no differences among ruminal gas production [31,32,78], some flavonoids (e.g., tannins) have effects on ruminal microbiota [17,68], modifying the gas production kinetics and the volatile fatty acids (VFA) proportions, sometimes improving the acetate: propionate ratio [17,41].

The chemical structure, distribution, and elimination of flavonoids affect the interaction and/or synergism between them and the ruminal microbiota.

Although there are many unknown ruminal interactions among some components of feedstuffs, microorganisms, and endogenous enzymes, anthocyanins could reduce the protozoa and microorganisms that may influence the rumen fermentation. However, feeding animals on residues with a high anthocyanin content as such berries seemed to have a low effect on fermentation patterns [44]. Some doses of proanthocyanidin may have a toxic effect (by altering the membranes’ permeability) on *Ruminococcus albus* and *Peptostreptococcus anaerobius*; meanwhile condensed tannins have a direct inhibitory effect on hemicellulases, endoglucanases, and proteolytic enzymes produced by *Fibrobacter succinogenes*, *Butyrivibrio fibrisolvens, Ruminobacter amylophilus,* and *Streptococcus bovis* [17]. Therefore, polyphenols have been associated with the reduction of fibrolytic enzymes and bacteria [17]. Moreover, some non-desirable antioxidant effects are the reduction of the endogenous fibrolytic enzymes activities and thereby the potential fiber digestibility and protein absorption [26].

Ruminal bacteria such as Anaerovibrio lipolytica, Butyrivibrio, Clostridium, Popionivacterium, Staphlylococcus, Selenomonas, and Pseudomonas aeruginosa have lipolytic activity, while Butyrivibrio fibrisolvens, Butyrivibrio hungatei, Clostridium strains, Propionibacterium, and Eubacterium participate in fatty acids biohydrogenation (BH) [17]. However, polyphenols could alter the ruminal microorganisms [79], altering some steps of BH. The lipolysis of dietary triglycerides, phospholipids, and glycolipids are sources of unsaturated fatty acids (UFA) to obtain stearic acid (C18:0) after sequential isomerization and saturation steps that involve the production of positional and geometrical isomers (C18:3, C18:2, C18:1 fatty acids). For example, regardless the type of polyphenol, in vitro studies have shown their negative effects on the growth of B. fibrisolvens that seem to lead to the accumulation of vaccenic acid and reduction of stearic acid [79]. There is not enough evidence clarify how specific changes in microbiota affect the BH.

The antioxidant activities of anthocyanin also affect increased desaturase enzymes activity for converting monounsaturated fatty acid (MUFA) to PUFA or inserting additional unsaturated bonds into already existing PUFA [17,27]. Studies have suggested that PUFAs have antimicrobial activities and are toxic to cellulolytic microorganisms by altering the bacterial cell membranes and the various essential processes that occur within and at the membrane; therefore, PUFAs can also reduce the microbial colonization with the fed particles and reduce the rumen digestibility of fiber [80].

In addition, as with other polyphenols sources, some HS components with high-lignin contents have low DM digestibility, and therefore, depending on the ruminant species and doses, the inclusion of by-products rich in polyphenols should not affect the DM intake or decrease the voluntary feed intake [26]. However, some authors suggest that adding certain antioxidants might not alter digestibility of DM, organic matter (OM), and neutral detergent fiber (NDF) [41], or even can reduce oxidative stress and increase the NDF, acid detergent fiber (ADF), and DM digestibility [40,41,80].

Novel molecular techniques (amplifying 16S rRNA) have shown that some flavonoids can increase the amount of *Bacteroidetes, Firmicutes*, and *Tenericutes,* and reduce the phyla *Proteobacteria, Verrucomicrobia,* and *Actonobacteria* [17].

The antioxidant and antimicrobial activities of HS are also related to the reduction of methane and N-*ammonia* (CH_4_ and NH_3_) caused by the changes of the by-products that affect the growth of methanogenic microorganisms [68]. Although it is not confirmed, different by-products rich in polyphenols (such as grapes, purple corn stover, Paulownia leaves, and other antioxidants) can affect the microorganisms participating in the production of hydrogen such as utilizing hydrogen to produce CH_4_ and therefore reduce it [17,40,41,44,45]. Thus, polyphenols can be associated with reductions of CH_4_ due to: (1) flavonoids indirectly reducing ruminal methanogenesis, acting as H_2_ sinks, (2) reduction of fiber digestion contributing to lower methane production, and (3) acting through the inhibition of the growth and activity of methanogens and hydrogen-producing microbes [17].

### 5.2. Post-Ruminal Effects of Anthocyanins

Some polyphenols are hydrolyzed and transformed through endogenous enzymatic activities and ruminal bacteria [81]; therefore, the secondary metabolites cross through the ruminal epithelium and the non-absorbed are bio-converted in the small bowel (as it occurs in monogastric) [81] and pass to the bloodstream [26,35,82,83] to deposit in tissues [68,82].

Anthocyanins can improve the blood plasma resistance to oxidation [33,84,85]. The COS and DOS can be deposited in lung, cardiac, renal, and hepatic tissues [69], suggesting that anthocyanins can improve the potential meat and milk antioxidants. However, improve of ruminal fatty acids biohydrogenation is associated with increased anthocyanins in animal products to human feed.

Although milk yield and fat have improvements related to anthocyanin addition in ruminant diets [85], the potential clinical effects of DOS and COS of HS on RAS could interfere in the homeostatic balance of ruminants and affect milk yield [84]. In mice, lactation led to upregulation and downregulation of selected RAS [86].

Reports about the potential effects of HS anthocyanins on fertility parameters are not consistent; however, other sources of polyphenols such as coffee can improve the semen quality [87] and reduce the fertilization rates even when progesterone, estradiol, and follicle-stimulating hormone remain constant [60]. In contrast, other types of antioxidants such as selenium and a-tocopherol might increase some reproductive parameters in dairy cattle [28]. Therefore, further studies could be focused on the effect of HS anthocyanins on estrous as well as milk and meat production.

Substituting 75% of total CP with HS seeds might not negatively affect animal performance in beef cattle [47]. Previously, the inclusion of ≤25% of the total DM of sheep diets with HS seeds increased the final body weight and carcass proportion [88]; however, in other studies, adding 10–20% of HS seeds improved the organoleptic and quality fatty acid properties of sheep meat [52].

## 6. Antioxidants Effect on Milk and Meat Quality as well as Shelf-Life

### 6.1. Anthocyanins and Polyphenols in Milk and Meat Fatty Acids Composition

Besides the positive effects of increasing the meat and milk antioxidants on human welfare, anthocyanins could increase the shelf-life of animal products [42,89,90]. Overall, polyphenols avoid lipid and protein oxidation (hyper-peroxides, aldehydes, and ketones), autolysis, and microbial pollution [29,91,92,93]. Increasing the long-chain fatty acids (n-3, n-6, and n-9) in ruminant diets improved the fatty acids composition of milk and meat [94,95,96].

Although there are not enough studies that analyze the effect of HS on milk fatty acids, among the literature, consistently including other products rich in polyphenols and anthocyanins increased the total PUFA, MUFA, and overall improved the milk fat C18:1, C18:2, *cis*-9, *trans*-11, n-3, n6, and concentrations of LA and conjugated linoleic acid (CLA) [26,41,44,45,79].

The antioxidant activity of milk and dairy products can be enhanced by phytochemicals rich in antioxidants [97]. Including by-products rich in polyphenols in dairy cows’ diets can increase the PUFA, lactose, and lactoglobulin in milk and its derived products, which have been associated with anticarcinogenic effects, and CLA and LA, which are associated with the reduction of atherogenic and thrombogenic indices [33].

In beef, by-products rich in polyphenols can alter the composition of fatty acids in meat through the effects of antioxidants on ruminal bacteria and the mechanism of absorption and transportation of these fatty acids from small intestine to muscle. However, some levels of antioxidants, primarily polyphenols and anthocyanins in ruminant diets, are associated with increases in the proportions of n-3 and n-6 PUFA [27]. Indeed, improving the contents of n-3 and n-6 PUFA in ruminant products have positive effects on human health, i.e., rumenic acid and LA isomers (*cis*-9, *trans*-11), which have demonstrated effects such as antiatherosclerosis, anticarcinogenic, antidiabetic, and anti-inflammatory activities in laboratory animals, and anticholesterolemic and anti-atherosclerosis effects in humans.

### 6.2. Anthocyanins and Polyphenols in Milk and Meat Shelf-Life

Antioxidants change the oxidative balance in dairy products and derivatives. Lipid oxidation is the main reason for the chemical spoilage of food and dairy products, decreasing their nutritional value, flavor, and texture.

Although milk proteins such as β and κ caseins have shown antioxidant activity, supplementation with different antioxidants such as vitamin C, tocopherol, vitamin E, zinc, and selenium can enhance the antioxidant activity of milk [97]. Recent reviews have been discussing the potential of using by-products of HS comparing their potential effects with other by-products rich in polyphenols and sources of anthocyanins (such as berries, grapes, grape pomace, etc.), to improve milk quality and fatty acids composition [26,33,41,44,45,79]. However, antioxidant compounds can also contribute to extending the shelf-life of dairy products and derivatives, reducing oxidative reactions that contribute to the deterioration of foods characterized by highly unsaturated lipids, which are extremely susceptible to oxidation [26], and reducing oxidized flavors in milk [98].

The fatty acid profiles of cheese have been shown to have the same variations as evidenced in milk. The quality of animal-derived foods is strongly associated with the characteristics of their lipid fractions [26]. Ianni et al. [33] monitored the extent of the oxidative damage in fresh and ripened cheeses through the evaluation thiobarbituric acid-reactive substances (TBARS), using malondialdehyde (MDA) as oxidative marker, and found that cheese from the milk of cows fed grape pomace did not increase MDA values 30 d after the ripening began, despite their greater contents of PUFAS vs. control (cows that did not feed on grape pomace) which went through oxidation. Grape pomace also reduced the concentrations of butanoic and hexanoic acids, associated with flavor changes and rancidity in both fresh and ripened cheeses. In addition, grape pomace increased aminobutyric acid (GABA) in cheese at the end of the ripening period that potentially can reduce the blood pressure, protect against chronic diseases, and improve immunity in consumers, but is also associated with specific fermentative bacteria (*Lactobacillus acidophilus* and *Lactobacillus hilgardii*) responsible for catalyzing the decarboxylation of l-glutamate to GABA.

Meat color, flavor, and odor are affected by oxidation which is shown as the conversion of red color muscle pigment myoglobin to brown metmyoglobin and the development of rancid odors and flavors [27].

In addition to the negative impact on meat pigments, odors, and flavors, lipid peroxidation causes toxic compounds implicated in several human pathologies, including aging processes, atherosclerosis, inflammation, and cancer [27]. Lipid oxidation in meat increases after 4 to 7 days of storage, and shelf-life and quality can be improved by natural antioxidants in meat by adding antioxidants in animal diets [98].

Protein-carbonyl determines protein oxidation in meat, and it increases during chilled storage. However, antioxidant and anthocyanin supplementation can slow increases in protein carbonyl during storage [27]. The HS by-products could have effects on meat lipid and protein oxidation comparable to other sources of by-products rich in anthocyanins, as previously studied [27,98,99,100].

In dairy cows, animals fed antioxidants improve the CLA and *cis*-9, *trans*-11 CLA, associated with the level of fatness. Feeding antioxidants in some stages of meat production can reduce the microbial growth and lipid oxidation during storage, increasing the shelf-life and meat quality [99]. Cattle fed other sources of antioxidants such as anthocyanins had a higher proportion of n-3 PUFA and show greater color stability and lower oxidation of lipid, protein, and myoglobin than meat from cattle fed with high oxidant diets (such as a high proportion of grains) [27].

Specifically, cattle fed antioxidants (primarily cyaniding-3-glucoside) had shown a more stable color depending on the dose of inclusion in diets. Maggiolino et al. [100] fed Merino lambs with lemon and red oranges rich in anthocyanins, finding that rheological, colorimetric, and oxidative parameters of *Longissimus lumborum* muscle sampled for 7 days were negatively affected by the time, but positively by the dose of anthocyanins supplemented. In this study, TBARS and hydroperoxides were also reduced, enhancing meat oxidative stability and improving the color in meet from lambs fed anthocyanins along the sampled period.

## 7. Limitations and Perspectives

The potential relationship among the antioxidant activities of calyxes, seeds, and stalks anthocyanins of HS with the ruminal microbiota and fibrolytic enzymes remain unknown. In comparison to the studies included in the present review that evaluated other polyphenols in the ruminal environment, hypothetically the positive effects of HS anthocyanins would be the potential reduction of CH_4_ and the fatty acids biohydrogenation process, but also it could reduce the potential fiber degradability [26,68,101,102,103].

Since antioxidants have a potential reduction of AOPP which is related with milk yield improvement, the available information about biochemical and RAS changes promoted by DOS and COS of HS [75,86] could be considered in further in vivo studies to find inclusion doses that would improve the composition of the antioxidant and fatty acids as well as improve milk yield. However, optimal inclusion should avoid potential negative effects on animal performance and reproductive parameters.

## 8. Conclusions

Including HS by-products might reduce the environmental and economic cost of livestock and potentially improve the quality of ruminant’s products. The excess of ROS unbalances the oxide-reductive potential primarily in ruminants fed. The HS contain flavonoids primarily classified as anthocyanins (mainly COS and DOS) that show specific actions on RAS regulation, increasing the ACEi action and reducing the expression of renin genes that could affect the animal productive behavior. As with other antioxidants, anthocyanins can reduce ruminal methanogens microorganisms and interact with substrates, fibrolytic microbiota, and enzymes affecting the fiber degradability and the biohydrogenation of lipids, improving the quality of milk and meat fatty acids composition; reducing the oxidation; improving the color, flavor, and odor stability; and extending the shelf-life of products. Changes in fatty acids composition can be beneficial for human consumers’ health.

## Figures and Tables

**Table 1:** *Hibiscus sabdariffa* L. chemical composition.

Authors	DM	CP	EE	CF	Ashes
	g/100 g	g/100 DM			
Fagbrnhro [2]	92.6	39.4	6.1	17.7	11.4
Maffo et al. [43]	90.0	22.0	22.0	20.0	6.1
Wang et al. [48]	NR	NR	18.0	NR	NR
Ismail et al. [49]	90.0	33.5	22.1	18.3	NR
Shaheen and El-Nakhlawy [50] *	NR	31.4	23.2	4.29	5.5
Udayasekhara [51] **	92.4	20.6	21.0	41.1	5.4
Beshir and Babikier [52]	96.6	30.3	11.1	5.1	5.6
Jínez et al. [53]	92.5	20.6	18.0	23.7	6.7
Kwari et al. [54]	NR	38.6	NR	13.5	NR
Mukhtar [55]	91.8	21.4	17.4	12.0	5.3
Soriano y Tejeda [56]	92.7	24.8	17.8	22.9	1.6
Anhwange et al. [57]	94.0	19.8	28.0	6.3	5.6
Tounkara et al. [58]	91.8	27.3	20.8	NR	4.5

DM, dry matter; CP, crude protein; EE, ether; CF, crude fiber; NR, not reported; * Average from three varieties; ** Average from two varieties.

**Table 2:** Proportion of fatty acids in *Hibiscus sabdariffa* L. seeds and calyxes.

	Seeds		Calyxes
	Tounkara et al. [58]	Mahmoud et al. [60]	Jabeur et al. [11]
Saturated fatty acids (%)			
Myristic (C14:0)	0.21	0.26	1.24 ± 0.01
Palmitic (C16:0)	19.21	20.52	27.73 ± 0.02
Stearic (C18:0)	5.13	5.79	4.46 ± 0.01
Arachidonic (C20:0)	0.67		1.02 ± 0.05
Polyunsaturated fatty acids (%)			
Palmitoleic (C16:1)	0.36		1.32 ± 0.04
Oleic (C18:1)	36.9	38.46	9.1 ± 0.1
Linoleic (C18:2)	35.02	33.25	32.65 ± 0.07
α-linoleic (C18:3)	1.85	1.69	15.76 ± 0.04

## Abbreviations

ACE, angiotensin converting enzyme; ACEi, inhibitor Angiotensin Converting Enzyme; ADF, acid detergent fiber; AMPK, AMP-dependent kinase; AngII, angiotensin II; AOPP, advanced oxidative protein products; BH, fatty acids biohydrogenation; CAM, calmodulin; CH4, methane; CLA, conjugated linoleic acid; CP, crude protein; CF, crude fiber; COS, cianidin-3-O-sambubioside; DM, dry matter; DOS, delphinidin-3-O-sambubioside; EGCG, epigalocathechin-3-gallate; EGC, epigallocatechin; ECG, epichatechin-3-gallate; EC, epicatechin; EGFR, epidermal growth factor receptor; GABA, aminobutyric acid. HS, *Hibiscus sabadariffa* L.; IGFR, insulin-like growth factor receptor; InsR, insulin receptor; I-κβ, interleukin; LA, linoleic acid; MDA, malondialdehyde; MUFA, monounsaturated fatty acids; NDF, neutral detergent fiber; NH3, N-ammonia; NF-κβ, necroptosis factor; OM, organic matter; PUFA, polyunsaturated fatty acids; SERCA, sarcoplasmic/endoplasmic reticulum Ca2+-ATPase activity; RAS, renin–angiotensin–aldosterone system; ROS, reactive oxygen species; RTK, receptor tyrosine kinase; SOD, superoxide dismutase; TBARS, thiobarbituric acid; TNF-α, tumoral factor; UFA, unsaturated fatty acids; VFA, volatile fatty acids.
